# Demographic, clinical, and radiological characteristics of cleidocranial dysplasia: A systematic review of cases reported in south America

**DOI:** 10.1016/j.amsu.2022.103611

**Published:** 2022-04-10

**Authors:** Eder Cano-Pérez, Claudio Gómez-Alegría, Fredy Pomares Herrera, Doris Gómez-Camargo, Dacia Malambo-García

**Affiliations:** a Grupo de Investigación UNIMOL, Facultad de Medicina, Universidad de Cartagena, Cartagena de Indias, Colombia; b Grupo de Investigación UNIMOL, Departamento de Farmacia, Facultad de Ciencias, Universidad Nacional de Colombia, Sede Bogotá, Bogotá, Colombia; c Grupo de Investigación en Atención Primaria en Salud/Telesalud, Facultad de Medicina, Universidad de Cartagena, Cartagena, Colombia; d Doctorado en Medicina Tropical, Facultad de Medicina, Universidad de Cartagena, Cartagena de Indias, Colombia

**Keywords:** Cleidocranial dysplasia, Clinical-radiological diagnosis, Craniofacial abnormalities, Skeletal abnormalities, South America

## Abstract

**Introduction:**

Cleidocranial dysplasia (CCD) is a rare disease characterized by craniofacial, skeletal, and oral anomalies. The disease prevalence is estimated to be 1 per million inhabitants; thus, only a few studies have described large cohorts of CCD patients. This study reviewed the clinical-radiological and demographic characteristics of patients with CCD in South America.

**Methods:**

We conducted a systematic review of all cases of CCD reported in South America following the PRISMA guidelines. Demographic information (sex, age at diagnosis, origin, reason for consultation, and family history) was also recorded. CCD signs were divided into “craniofacial” and “skeletal” categories.

**Results:**

A total of 72 cases were included. We found that oral anomalies were the most common reason for consultation leading to a diagnosis in patients, with a median age at diagnosis of 14 years. Fifty percent of the patients were women. Open fontanels or cranial sutures, the presence of at least one of the typical CCD facies (frontal bossing, brachycephaly, hypertelorism, or depression of the nasal bridge), and supernumerary teeth were reported in 92%, 85%, and 88% of cases, respectively. Clavicular dysplasia was present in 98.6% of cases, and other skeletal abnormalities such as scoliosis, pubic symphysis diastasis, and flat feet were found; short stature was present in 71% of cases, and one case presented cognitive deficits.

**Conclusion:**

Although the phenotypic spectrum of CCD is variable, clavicular dysplasia, open fontanels or cranial sutures, dental anomalies, and at least one of the typical CCD facies are present in at least 80% of cases.

## Highlights

• About 70% of patients are diagnosed during childhood or adolescence, mostly due to dental complications.
• Clavicular dysplasia, open fontanel, dental abnormalities, and facies typical of CCD are present in at least 80% of cases.
• The prevalence of CCD signs in the South American population is similar to other patient groups from other populations.

## 1 Introduction

Cleidocranial dysplasia (CCD) is an autosomal dominant disease characterized by the presence of craniofacial, skeletal, and oral anomalies, including clavicular aplasia or hypoplasia, delayed closure of fontanels, midface hypoplasia, brachycephaly, supernumerary teeth, and short stature [1]. The prevalence of this condition is estimated to be 1 per million inhabitants, with no ethnic- or sex-associated predisposition [2,3]. However, the disease is likely underdiagnosed owing to the relative absence of serious health complications for the affected person when compared with those of more severe skeletal dysplasias [3].

CCD is caused by mutations in the *Runx2* gene (located at the locus 6p2143), which encodes a transcription factor that activates osteoblast differentiation and skeletal morphogenesis [4,5]. Several nonsense, antisense, and frameshift mutations that cause haploinsufficiency in the RUNX2 protein and chromosomal translocations and deletions that lead to loss of the entire gene have been identified [[5], [6], [7]].

Despite the autosomal dominant nature of CCD, the phenotypic characteristics of the disease are variably expressed between individuals and even within the same family group, with phenotypic spectra ranging from mild cases presenting with supernumerary teeth to cases with severe defects in skeletal development [1,8]. However, the clinical-radiological characteristics of CCD are commonly described in case reports, familial case series, and relatively small single-center cohorts. Few studies have reviewed the prevalence of the characteristic signs of the condition in a large number of patients. This study reviewed the CCD cases reported in South America and described the clinical-radiological features of the disease based on the scenario in South America.

## 2 Methods

This review followed the PRISMA (Preferred Reporting Items for Systematic Reviews and Meta-Analyses) 2020 statement (Supplementary file 1) [9]. The quality of this systematic review was assessed using the AMSTAR 2 criteria (Assessing the Methodological Quality of Systematic Reviews) and was found to be of moderate quality (Supplementary file 2) [10]. This study was registered at www.researchregistry.org with identification number reviewregistry1292.

### 2.1 Information sources and search strategy

A directional search with the keywords “cleidocranial dysplasia”, “cleidocranial dysostosis”, and “Pierre Marie-Sainton syndrome” accompanied by the terms “Latin America”, “South America”, or the names of each country in the region (Argentina, Bolivia, Brazil, Chile, Colombia, Ecuador, Paraguay, Peru, Uruguay, and Venezuela) were performed in PubMed, Scopus, and Embase, as well as alternative sources such as Google Scholar, SciELO, and LILACS. References to the consulted articles were also reviewed. The bibliographic search was carried out until December 2021, and manuscripts written in English, Spanish, and Portuguese were considered, and no limit was put on the year of publication of the studies.

### 2.2 Selection of studies and eligibility criteria

Two authors (ECP and DMG) independently searched for and reviewed each potentially eligible article. Case and case series studies that described the demographic and clinical-radiological characteristics of patients as completely as possible and individually (for case series studies) were evaluated. Reports that only provided oral descriptions, cohort studies without individual patient information, reports focusing on specific pathological findings where the CCD condition was not the main interest, clinical exercise articles, and reports that did not have full text were excluded.

### 2.3 Data extraction

The data were extracted and tabulated in Excel. Among the demographic information, the following variables were recorded: sex, age at diagnosis, origin, reason for consultation, and family history. Signs and symptoms were divided into “craniofacial” and “skeletal” categories. Craniofacial signs included typical CCD facies, such as frontal bossing, brachycephaly, hypertelorism, and nasal bridge depression. Midface hypoplasia, open fontanels or cranial sutures, presence of Wormian bones, late eruption of secondary teeth, late exfoliation of deciduous teeth, and supernumerary teeth (erupted or unerupted) were also evaluated. Skeletal abnormalities included clavicular dysplasia confirmed by radiography or by the clavicular sign (ability to approximate the shoulders to the thoracic midline) as an indicator of dysplasia in cases without radiography, as well as spinal, pelvic, hand, and foot anomalies. Short stature was also included in this category. Because the description of cases in the literature was not always complete, we refrained from naming other skeletal disorders. The presence of mutations in *Runx2* was recorded whenever possible.

### 2.4 Data analysis

A pooled analysis of the patients was performed to assess their demographics and clinical-radiological characteristics. Only cases for which information was available were considered for the analysis of each sign or variable. Descriptive statistics were generated. Comparisons between continuous variables were performed by Mann-Whitney *U* test using the SPSS v.19.0 software (IBM Corp, Armonk, USA), with statistical significance being defined at p < 0.05.

## 3 Results

### 3.1 Systematic review

**Fig. 1:**
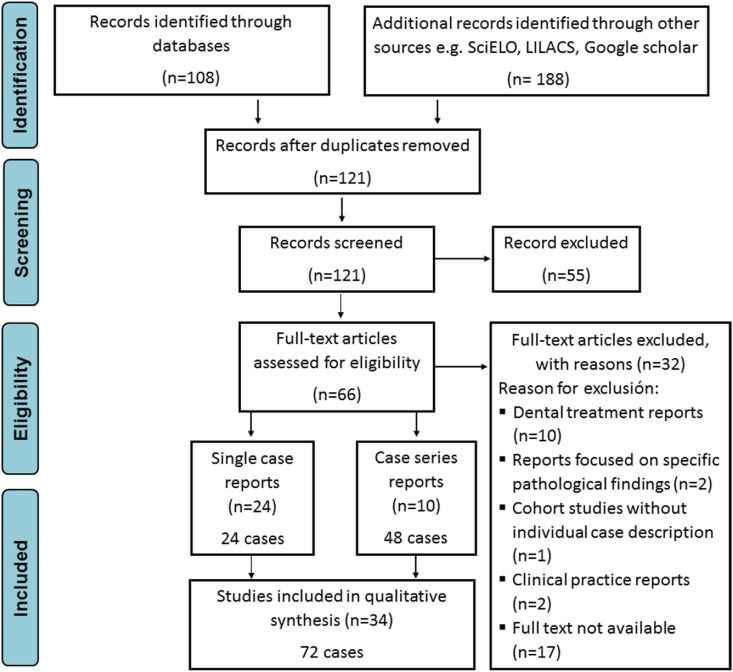
Selection of the cleidocranial dysplasia cases included in the study according to the PRISMA guidelines.

Through the search protocol described above, 296 articles in English, Spanish, or Portuguese were identified, of which 230 were excluded as duplicates, reports from outside South America, or non-reports of CCD. 66 full-text papers were assessed for eligibility before inclusion, of which 32 were excluded for different reasons. Finally, 34 manuscripts were included in the study, which were classified into 24 single case presentations [[11], [12], [13], [14], [15], [16], [17], [18], [19], [20], [21], [22], [23], [24], [25], [26], [27], [28], [29], [30], [31], [32], [33], [34]] and 10 case series reports [[35], [36], [37], [38], [39], [40], [41], [42], [43], [44]], representing 48 patients among a total of 72 cases included in this study (Fig. 1).

### 3.2 Geographical distribution of cases

**Fig. 2:**
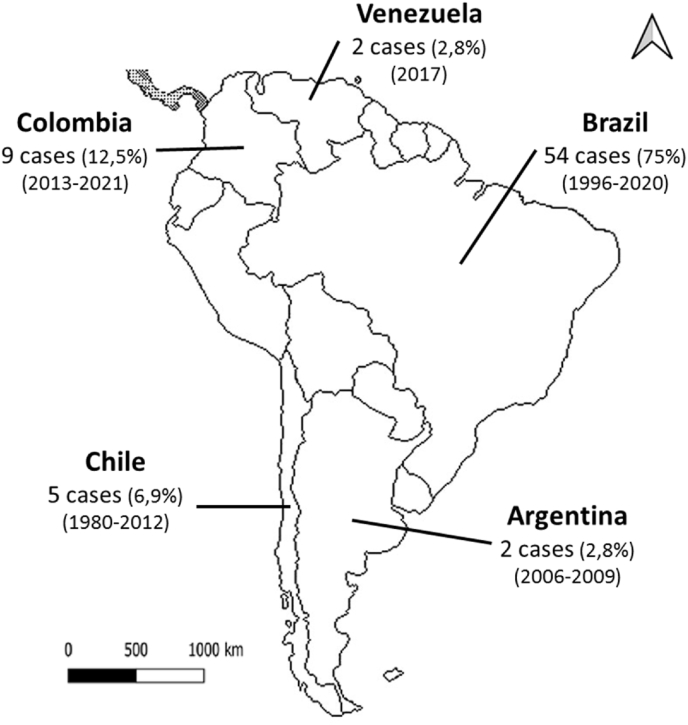
Geographic distribution of cleidocranial dysplasia cases reported in South America.

Fig. 2 presents the geographic distribution of the 72 cases of CCD reported in South America. The patients came from five countries in the region, with Brazil having the most reports, thus contributing 75% (n = 54) of the CCD case presentations. Reports from Colombia (nine cases), Chile (five cases), Argentina (two cases), and Venezuela (two cases) accounted for the remaining 25%.

### 3.3 Sex and age

The sex distribution of the patients was equal between males (n = 36) and females (n = 36). Overall, the median age at diagnosis was 14 years (range, 0–84), however, a statistical difference in age at diagnosis was observed between females and males [females, 19.5 years (0−84) vs. males, 11 years (0−46), p = 0.002]. Fifty-three (74.6%) patients were diagnosed before the age of 25 years, while 18 (25.4%) were diagnosed late in adulthood or old age. The distribution of the cases according to age and sex is shown in Table 1. The age of only one male patient was not specified.

**Table 1:** Distribution of the age of cleidocranial dysplasia patients at diagnosis by stage and sex.

Age at diagnosis					
Stage	Age range (years)	Number of cases			%
		M^a^	F	Total	
**Infants**	0–5	7	5	12	16.9
**Children**	6–11	11	6	17	23.9
**Adolescent**	12–20	12	8	20	28.2
**Young**	21–25	2	2	4	5.6
**Adult**	26–60	3	13	16	22.5
**Old age**	>60	0	2	2	2.8
Total		35	36	71	100

a The age of one male patient was not specified.

### 3.4 Family history

Among the 24 probands in the single-case presentations, eight did not report family history, 10 did not present family information relevant to the condition, and six cases presented with parents and/or siblings with CCD. Of the 48 cases with multiple presentations, six were sporadic, and 42 were seen in 15 families.

### 3.5 Clinical and radiological manifestations

Of the total number of cases, 47 (65.3%) reported reasons for consultation. The two main reasons were oral anomalies (late exfoliation of deciduous teeth, late eruption of secondary teeth, supernumerary teeth, or toothache) in 44.7% (21/47) of the patients and cranial-skeletal anomalies (clavicular agenesis, open fontanels, or facial dysmorphia) in 29.8% (14/47) of the patients. Other reasons included short stature, findings due to neonatal control, and general consultation. Some patients had more than one reason for consultation.

Table 2 summarizes the clinical and radiological findings from the patients. Among the craniofacial anomalies, fontanels or open cranial sutures were reported in 92.6% of the cases and the presence of Wormian bones was reported in 90.3% of the patients who had a skull radiograph. 85% of patients presented with at least one of the typical CCD facies during physical examination, with frontal bossing and hypertelorism being the most frequent, in 67.6% of cases each. There was a delay in the eruption of secondary teeth in 78.8% of the patients aged between 5 and 84 years, and 88% had supernumerary teeth that were erupted or revealed by radiography.

**Table 2:** Summary of the clinical-radiological findings of cleidocranial dysplasia cases reported in South America and comparison with other groups of patients from other populations.

Clinical-radiological manifestations	**South America (this study)**	Other groups of CCD patients				
		USA [51]	Middle Europe [52]	Poland [49]	Turkey [50]	Turkey [54]
**Craniofacial abnormalities, *n* (%)**						
**Open fontanels or cranial sutures**	50/54 (92.6)	22/33 (66.7)	NA	12/12 (100)	14/15 (93.3)	39/44 (88.6)
**Presence of Wormian bones**	28/31 (90.3)	26/33 (78.8)	21/22 (95.4)	10/12 (83.0)	NA	19/37 (51.3)
**Brachycephaly**	33/71 (46.5)	NA	NA	NA	NA	8/20 (40.0)
**Frontal bossing**	48/71 (67.6)	NA	23/25 (92.0)	NA	15/15 (100)	41/51 (80.4)
**Hypertelorism**	48/71 (67.6)	27/31 (87.1)	NA	10/12 (83.0)	15/15 (100)	37/51 (72.5)
**Nasal bridge depression**	27/71 (38.0)	NA	24/26 (92.3)	NA	NA	NA
**Midface hypoplasia**	26/60 (43.3)	NA	25/26 (96.1)	10/12 (83.0)	13/15 (100)	48/51 (94.1)
**Late exfoliation of deciduous teeth**	16/27 (59.3)	NA	NA	NA	NA	41/49 (83.7)
**Late eruption of secondary teeth**	41/52 (78.8)	NA	25/26 (96.1)	NA	8/8 (100)	37/50 (74.0)
**Supernumerary teeth**	44/50 (88.0)	29/39 (74.3)	24/26 (92.3)	6/8 (75.0)	7/7 (100)	17/44 (38.6)
**Skeletal abnormalities, *n* (%)**						
**Clavicular dysplasia**	71/72 (98.6)	40/40 (100)	22/26 (84.6)	12/12 (100)	13/13 (100)	36/38 (95.0)
**Both hypoplastic clavicles**	41/71 (57.7)	23/40 (57.5)	NA	NA	NA	NA
**Both aplastic clavicles**	17/71 (23.9)	0 (0)	NA	NA	NA	NA
**Aplastic/hypoplastic**	5/71 (7.0)	0 (0)	NA	NA	NA	NA
**Aplastic/normal**	1/71 (1.4)	17/40 (42.5)	NA	NA	NA	NA
**Dysplasia defined by clavicular sign**	7/71 (9.9)	0 (0)	22/22 (100)	NA	NA	NA
**Pelvic abnormalities**	18/28 (64.3)	17/29 (58.6)^a^	NA	10/12 (83.0)	NA	NA
**Spinal abnormalities**	7/19 (36.8)	27/40 (67.5)^b^	NA	NA	2/14 (14.3)	14/50 (28.0)
**Hand and/or feet anomalies**	21/31 (67.7)	22/24 (91.7)	NA	NA	11/12 (91.7)	21/51 (41.1)
**Short stature**	40/56 (71.4)	NA	6/26 (23.0)	11/12 (92.0)	4/15 (26.7)	22/51 (43.1)
**Cognitive deficit**	1/67 (1.5)	NA	NA	NA	NA	NA
**Mutation Presence (*Runx2*)**	11/13 (76.9)	NA	18/18 (100)	4/7 (57.1)	14/15 (93.3)	44/44 (100)

NA: not available.

a Only cases with iliac wing hypoplasia.

b Only cases with spina bifida.

With regard to skeletal abnormalities, 98.6% (71/72) of the patients manifested clavicular dysplasia; of these, approximately 90% (n = 64) were confirmed by radiological studies and 10% (n = 7) were defined by clavicular signs. Hypoplasia of both clavicles was present in 57.7% of cases, whereas aplasia of both clavicles was present in 23.9% of cases. In contrast, five individuals presented with clavicle hypoplasia/aplasia, another presented with right clavicle aplasia and a normal left clavicle, and one patient presented with normal clavicles. Additional skeletal findings were described in the patients, such as scoliosis, pubic symphysis diastasis, hypoplasia of the distal phalanges of the hands, and flat feet. Short stature was present in 71.4% of patients, and one patient presented cognitive deficit. Molecular analyses were performed in 14/72 cases, of which two were sporadic, nine were familial, and in three members of one family, it was not possible to detect the mutation (Table 3).

**Table 3:** Summary of mutations in the *Runx2* gene reported in cleidocranial dysplasia cases from South America.

Family (number of affected individuals)	Family history	Mutation					Reference
		Nucleotide	Amino acid	Exon	Type	Domain	
**1 (1)**	De novo	674G > A	R225Q	3	Missense	Runt	[11]
**2 (2)**	Familial	674G > A	R225Q	3	Missense	Runt	[35]
**3 (1)**	De novo	674G > A	R225Q	3	Missense	Runt	[36]
**4 (2)**	Familial	569C > T	R190Q	2	Missense	Runt	[36]
**5 (2)**	Familial	674G > A	R225Q	3	Missense	Runt	[36]
**6 (3)**	Familial	Q292fs fi X299	873_874delCA	5	Frameshift	PST	[36]
**7 (3)**	Familial	ND	–	–	–	–	[36]

ND, not detected.

## 4 Discussion

This study described the demographic and clinical–radiological characteristics of 72 cases of CCD reported in South America. This constitutes the first systematic analysis of this condition in the region and one of the few studies that describes the characteristics of CCD in a large group of patients, considering the low prevalence of this rare disease.

We found that dental complications were one of the main factors that resulted in patient diagnosis because failed exfoliation of deciduous teeth during childhood or late eruption of secondary teeth in adolescence motivated dental consultation, leading to the diagnosis of CCD. This is consistent with the median age at diagnosis of 14 years, suggesting that the condition is usually discovered during childhood/adolescence [[45], [46], [47]]. However, 25% of patients were diagnosed in adulthood or old age, possibly due to the relative absence of serious health complications in the early lives of patients [48]. Early diagnosis is important to act appropriately on disorders that may occur later, such as skeletal dysplasia and dental complications; these may affect the self-esteem and social interaction of the adolescents. Therefore, intervention by a multidisciplinary health team is recommended in all cases.

Clavicular dysplasia was the most reported skeletal feature used to establish the diagnosis, which presented in 98.6% of the cases, and was considered the most frequent characteristic in the patients. This characteristic has been reported in 100% of cases in some radiological studies [45,[49], [50], [51]] and between 84 and 97% of cases during physical evaluations [47,52]. Furthermore, the clavicles are usually hypoplastic or discontinuous in patients with CCD, either unilaterally or bilaterally, while clavicles are completely absent in approximately 10% of cases [1]. In this study, hypoplasia of both clavicles was the most frequently found type of dysplasia (57.7%), similar to that reported by Jarvis et al. [51] and Yoshida et al. [53], in which hypoplasia of both clavicles was present in 57.5% and 75% of patients, respectively. In contrast, one patient presented with two normal clavicles; however, other craniofacial and skeletal anomalies, as well as molecular studies, confirmed their CCD diagnosis [12]. Other cases of CCD with normal clavicles have been reported in the literature [54]. In addition to the clavicles, other orthopedic problems in CCD patients involve the hands, feet, pelvis, and spine [55]. In this study, spinal abnormalities, such as scoliosis, occurred in five patients. In the hands and feet, hypoplasia of the distal phalanges and flat feet were the most common abnormalities, while in the pelvis, pubic symphysis diastasis was the most frequent. Studies have reported a higher prevalence of anomalies, such as scoliosis and flat feet, in 17% and 57% of CCD cases, respectively [3,54]. Conditions such as scoliosis may require surgical treatment because spinal deformities in CCD are progressive.

With regard to craniofacial anomalies, we found that most cases with available skull radiographs presented with Wormian bones and open fontanels or cranial sutures (90–92% of cases), consistent with reports in groups of patients with CCD from Central Europe, Poland, and Turkey (Table 2). Other features, such as frontal bossing and hypertelorism, were the most reported typical CCD facies, but with relatively lower frequencies than those of the other patient groups, and the frequency of dental anomalies, such as supernumerary teeth, was comparable to that of other studies [47] (Table 2). Although the phenotypic spectrum of CCD is variable even among relatives, our data suggest that overall, clavicular dysplasia, open fontanels or cranial sutures, dental anomalies, and at least one of the typical CCD facies are present in at least 80% of CCD cases.

Further, CCD is usually not associated with cognitive deficits. However, a patient with a learning disability and cognitive deficit was found here [30], which has been previously reported in other CCD cases [56,57]. Moreover, 71% of the patients with available data presented short stature. Significantly shorter stature has been reported in men and women with CCD than in the subjects from control groups; however, short stature due to CCD may not be severe enough to categorize the disease as a dwarfism-associated condition [3].

The *Runx2* gene encodes the RUNX2 transcription factor, which activates osteoblast differentiation and skeletal morphogenesis. Mutations in this gene cause haploinsufficiency of the protein [5]. Generally, mutations occurring within the Runt domain result in a classic CCD phenotype. However, due to the widely variable phenotypic expression in CCD patients, conclusive genotype-phenotype correlations have been difficult to establish [58]. Nevertheless, short stature and dental problems are significantly milder in patients with mutations outside the Runt domain than in patients with mutations within the domain [53,58]. However, a recent study found no significant association between patient stature and alterations in this domain [54]. In the current study, only 14 of the 72 cases underwent molecular analyses of the *Runx2* gene, of which 11 had mutations. Interestingly, eight cases with mutations and three cases without mutations were described in a study that reported that cases with mutations in the Runt domain showed a higher number of impacted and supernumerary permanent teeth than those without mutations in the Runt domain or the *Runx2* gene [36]. The low frequency of molecular studies used in the cases presented herein may reflect the important role of clinical and radiological studies in establishing the diagnosis of CCD according to the pathognomonic signs of the disease. However, when the clinical or radiological diagnosis is in doubt, it is advisable to analyze mutations in the *Runx2* gene.

One limitation of this study is that the case reports and series were often not described extensively or in detail, which restricted the overall analysis of some features of the disease. Despite these limitations, to the best of our knowledge, this study is the most extensive description of CCD in the region till date; it provides a foundation for future studies regarding this condition.

## 5 Conclusions

CCD is a generalized skeletal dysplasia that primarily affects the bones of the axial skeleton. Although the phenotypic spectrum of the disease is variable, our data suggest that clavicular dysplasia, open fontanel and cranial sutures, dental abnormalities, and facies typical of CCD are present in at least 80% of cases. Although CCD is associated with various skeletal anomalies, our findings suggest that most cases are diagnosed based on dental consultation, with the diagnosis being established mainly in childhood and adolescence. Likewise, considering the distinctive clinical signs of CCD, it is essential, and in many cases, sufficient, to use clinical–radiological studies for the diagnosis of the condition. However, analysis of mutations in the *Runx2* gene is recommended in cases that require molecular confirmation. Finally, although there is no curative therapy for CCD complications, it is possible to plan and implement a multidisciplinary treatment aimed at improving the quality of life of patients with this condition.

## Sources of funding

None.

## Ethical approval

Ethical approval was not required.

## Consent

None.

## Authors contribution

Eder Cano-Pérez - study concept or design, data collection, data analysis or interpretation, writing the paper.

Claudio Gómez-Alegría – data analysis or interpretation, edition and final approval.

Fredy Pomares Herrera – data interpretation, edition and final approval.

Doris Gómez-Camargo – data interpretation, edition and final approval.

Dacia Malambo-García - study design, data collection, data analysis or interpretation, writing the paper, and final approval.

## Registration of research studies

1. Name of the registry: Research Registry
2. Unique Identifying number or registration ID: reviewregistry1292
3. Hyperlink to your specific registration (must be publicly accessible and will be checked): https://www.researchregistry.com/browse-the-registry#registryofsystematicreviewsmeta-analyses/registryofsystematicreviewsmeta-analysesdetails/61fafa5e33d019001fa5b11d/

## Guarantor

Eder Cano-Pérez and Dacia Malambo-García.

## Declaration of competing interest

The authors have no conflicts of interest to declare.
